# Natural eggshell membrane supplementation for chronic lameness in warmblood horses: a 12-week prospective before–after study

**DOI:** 10.3389/fvets.2026.1711135

**Published:** 2026-02-26

**Authors:** Young-Sam Kwon, Hyohoon Jeong, Jongkyu Kim, Jina Kim, Kyungmin Chun, Sung Keun Yang, Byungkwon Kim

**Affiliations:** 1 Department of Veterinary Surgery, College of Veterinary Medicine, Kyungpook National University, Daegu, Republic of Korea; 2 Large Animal Clinical Medicine, College of Veterinary Medicine, Jeju National University, Jeju-si, Republic of Korea; 3 Division of Clinical Trial Management, Affiliated Research Institute, Ari B&C Co., Ltd., Yongin-si, Republic of Korea; 4 ACE Equestrian Team, Cheongdeok High School, Yongin-si, Republic of Korea; 5 ACE Equestrian Team, Vivian & Stanley Gangnam International Scholars, Seoul, Republic of Korea; 6 Affiliated Research Institute, Seongdong Bio Co., Ltd., Goyang-si, Republic of Korea; 7 College of Korean Medicine, Wonkwang University, Iksan-si, Republic of Korea

**Keywords:** equine osteoarthritis, joint range of motion, kinematic assessment, lameness evaluation, natural eggshell membrane, nutraceutical supplementation rider-reported outcomes

## Abstract

**Introduction:**

Osteoarthritis is a leading cause of equine lameness, yet pragmatic evidence for nutraceuticals in horses remains limited.

**Methods:**

We prospectively evaluated 12 weeks of daily natural eggshell membrane (NEM; 12 mg/kg, orally) supplementation in Warmblood horses with chronic lameness using a single-arm before–after design. Ten horses were enrolled and prespecified paired contrasts compared visit 3 (V3, week 12) with baseline (V1). Outcomes included rider-reported under-saddle function (walk and trot), examiner-graded lameness (rest and walk–trot composite), simple joint-angle kinematics (degrees), and owner-rated palatability.

**Results:**

Rider-reported function improved by approximately half a grade at both walk and trot, and owner-rated palatability improved markedly. Examiner walk-trot scores showed a small trend toward improvement, while rest lameness remained unchanged. Statistical inference supported these patterns, with 95% confidence intervals (CIs) excluding zero for rider scores (Δ = −0.50) and palatability (Δ = −1.50). Moderate changes were observed in right-fore joint angle (Δ = +3.06°) and examiner composite grades (Δ = −0.11). These effects were supported by small-sample inference (permutation tests, bootstrap CIs) and complementary Bayesian estimation.

**Discussion and conclusion:**

NEM showed potential short-term improvements in owner-reported function and examiner-graded gait, but larger controlled studies are needed.

## 1 Introduction

Osteoarthritis (OA) is the most frequent cause of chronic lameness in horses and a major contributor to reduced performance, welfare concerns, and shortened athletic careers (1–3). Joint disease is estimated to account for the majority of equine lameness, with OA affecting more than half of older horses and contributing to approximately 60% of lameness presentations overall (2, 3).

Clinical lameness is typically graded on the American Association of Equine Practitioners (AAEP) 0–5 scale. While the standard AAEP lameness scale facilitates clinical communication, its observer dependence can limit sensitivity to modest functional change (1, 4–6). Consequently, triangulating these grades with rider-reported outcomes and objective kinematics offers a more robust strategy for detecting modest improvements in previous studies (5, 6).

Conventional radiography is widely used to monitor OA, but radiographic surrogates of cartilage loss such as apparent joint-space width (JSW) can be difficult to interpret over short intervals because they are sensitive to imaging technique and positioning. As a result, small structural changes may be challenging to distinguish from technical variation, and structural imaging alone may not fully capture clinically relevant improvement. Many clinical programs, therefore, combine examiner-graded lameness with rider-reported function and simple kinematic measures (e.g., joint angles) to better reflect day-to-day performance (5, 7).

Natural eggshell membrane (NEM) is a concentrated source of joint-relevant biomolecules, including collagen, glycosaminoglycans, hyaluronic acid, which can contribute to cartilage matrix support, lubrication, and shock absorption. Preclinical studies also indicate that NEM exerts anti-inflammatory and chondroprotective effects, including modulation of proinflammatory cytokines implicated in OA pain and stiffness and attenuation of cartilage-degrading processes (8–10).

In humans with knee OA, randomized and controlled trials have reported clinically meaningful reductions in pain and stiffness within 1 week–12 weeks of NEM supplementation, with favorable safety profile (8, 10–13). Veterinary and experimental animal studies similarly show improvements in mobility and quality-of-life measures, as well as chondroprotection, with an eggshell-membrane–containing formulations (14–17). Although equine-specific evidence remains limited, NEM has been incorporated into equine joint supplements and investigated alongside chelated trace minerals in mixed-species programs, supporting translational plausibility to horses (16, 18). Consequently, pragmatic trials are now required to assess the feasibility, acceptability, and functional impact of NEM in horses under field conditions.

Despite this background, there are few data directly evaluating NEM in horses with naturally occurring lameness using paired clinical and functional outcomes. To address this gap, we conducted a 12-week, single-arm before–after study in Warmblood horses with chronic lameness. We assessed examiner-graded lameness (AAEP-aligned scales), rider-perceived efficacy at walk and trot, and simple joint-angle kinematics. Palatability matters for long-term oral dosing because voluntary intake is a major determinant of acceptability and owner compliance in veterinary patients (19). We also included owner-reported palatability as a feasibility endpoint reflecting acceptability and ease of administration to capture both functional response and feasibility of sustained dosing. We hypothesized that NEM would be well tolerated, would improve rider-perceived function, and would show supportive trends in joint-angle kinematics over 12 weeks.

## 2 Materials and methods

### 2.1 Ethical approval

All procedures required for this present study were approved and conducted by the Institutional Animal Care and Use Committee (IACUC) of Kyungpook National University (Approval Number: KNU 2025–0401). Informed written owner consent and the use of data for scientific purposes were obtained prior to enrollment. The study adhered to relevant institutional and national guidelines for the care and use of animals.

### 2.2 Study design

#### 2.2.1 Design and scheduling

This was a prospective, single-arm before–after pilot conducted over a 12-week period in privately owned Warmblood horses with chronic lameness. Additionally, this was an open-label pilot; thus, riders and owners were aware that horses were receiving NEM, but they were not given specific expectations regarding timing or magnitude of effect. The same rider evaluated each horse at all visits whenever feasible to reduce measurement variability and preserve within-horse consistency.

The study timeline included three assessment points:

- Baseline (Visit 1, V1): Initial assessment prior to supplementation.
- Week 4 (Visit 2, V2): An intermediate assessment used for descriptive visualization only.
- Week 12 (Visit 3, V3): The primary endpoint for efficacy evaluation. Inferential analyses for hypothesis tests were restricted to paired comparisons between V1 and V3.

At each visit, horses were ridden by their usual rider (where feasible) following a standardized pattern consisting of walk and trot in straight lines and 20 m circles in both directions in a familiar arena. Riders were instructed to focus on overall comfort, willingness to move forward, ease of transitions, and perceived stiffness or resistance under saddle.

#### 2.2.2 Dosing and administration

Horses received natural eggshell membrane (NEM) supplied by Seongdong Bio Co., Ltd. (Republic of Korea). NEM was provided as an odorless, flavor-neutral powder mixed with the horse’s usual concentrate. The supplement was administered orally once daily at a dosage of 12 mg/kg for the full 12 weeks. Owners mixed the calculated daily amount into the ration immediately prior to feeding and maintained a daily log to document the body weight, the administration date and time, and any dosing deviations.

#### 2.2.3 Dosing and administration

To isolate the intervention effect, concomitant analgesics and intra-articular therapies were discouraged unless clinically necessary. Any administration of such rescue medication use was recorded.

### 2.3 Animals, eligibility, and study endpoints

#### 2.3.1 Animals and eligibility

Inclusion criteria required horses to be Warmbloods aged ≥7 years with chronic lameness persisting for at least 3 months. Eligible horses presented with mild-to-moderate forelimb lameness (AAEP grades 1–3/5) at the trot on a hard surface, localized by veterinary examination. Owners provided written consent to maintain stable housing and routine activity levels (whether actively or lightly ridden) throughout the 12-week study.

Horses were excluded if they presented with acute trauma, laminitis, severe neurological deficits, or life-threatening systemic disease (e.g., organ failure). To ensure joint-specific efficacy could be evaluated, horses with primary lameness localized to the shoulder or hind limbs were excluded, although secondary compensatory gait abnormalities were permitted.

A strict washout period was enforced to minimize confounding variables. Horses were excluded if they had received intra-articular corticosteroids (e.g., triamcinolone acetonide, betamethasone) or hyaluronic acid within 6 weeks prior to baseline (V1). Specifically, seven horses with a history of prior steroid treatment underwent a mandatory 6-week washout, while the remaining three underwent a concurrent 6-week acclimatization period. The administration of other joint-targeted nutraceuticals or NSAIDs was prohibited unless the regimen could be clinically justified and maintained at a constant stable dose. In this cohort, however, all such modulators were withdrawn during the washout.

Following veterinarian confirmation at screening, 10 male Warmblood horses with a mean age of 14.9 ± 2.18 years were enrolled. To minimize inter-rater variability, horses remained under usual management, and where feasible, the same veterinarian, rider, and owner assessor were retained across visits. Detailed baseline signalment, work status, and localized lameness diagnoses for the enrolled horses are summarized in Table 1.

**Table 1:** Demographic characteristics, activity status, and baseline clinical diagnoses of the study cohort.

ID	Sex	Age	Breed	Reproductive status	Primary discipline/Job classification	Activity/Retirement status	Localized lameness site	Multi-limb lameness status
H01	M	12	Warmblood	Gelding	Ex–show jumper/Schooling jumper	Lightly ridden	No chronic lesion; mild distal limb discomfort only	Multi-limbs
H02	M	12	Warmblood	Gelding	Show jumper (active sport horse)	Actively ridden	LF coffin joint inflammation (primary at baseline) and prior RF navicular changes	Multi-limbs
H03	M	13	Warmblood (Dutch)	Gelding	Show jumper/Schooling jumper	Actively ridden	RF coffin joint OA and navicular syndrome	Multi-limbs
H04	M	15	Warmblood (Hanoverian)	Stallion	Show jumper (stallion class)	Lightly ridden	LF ring bone (PIP joint OA)	Single limb
H05	M	17	Warmblood (Belgian)	Gelding	Ex–show jumper/Senior schooling horse	Lightly ridden/Semi-retired	RF medial side bone (collateral cartilage ossification)	Single limb
H06	M	19	Warmblood	Gelding	Ex–show jumper/Companion/Schooling	Semi-retired	RH deep digital flexor tendon strain	Single limb
H07	M	15	Warmblood (Holsteiner)	Gelding	Show jumper/Schooling jumper	Actively ridden	LF fetlock joint OA	Single limb
H08	M	15	Warmblood	Gelding	Show jumper	Actively ridden	RH hock joint OA	Single limb
H09	M	15	Warmblood	Gelding	Show jumper/Schooling jumper	Actively ridden	RF navicular syndrome	Single limb
H10	M	16	Warmblood (Dutch)	Gelding	Ex–show jumper/Schooling jumper	Lightly ridden	LH medial side bone	Single limb

LF, left fore; RF, right fore; LH, left hind; RH, right hind; OA, osteoarthritis; PIP, proximal interphalangeal.

#### 2.3.2 Study endpoints

Clinical, rider-reported, kinematic, and acceptability endpoints were prespecified. For all measures, the primary contrast was defined as the change (Δ) from baseline (V1) to week 12 (V3): Δ = V3 − V1. Endpoints were defined as follows:

- Examiner-graded lameness: Assessed at rest and as a walk–trot composite using ordinal scales aligned with AAEP practice (lower scores = better).
- Rider efficacy: Evaluated at walk and trot using study-defined ordinal anchors to capture under-saddle function (lower scores = better). Under-saddle function was rated on a 0–5 ordinal scale at walk and trot (0 = no perceived impairment; 5 = marked difficulty performing the pattern). Scale anchors and brief descriptors were provided on a standardized form completed immediately after the ride.
- Joint-angle kinematics: Measured in degrees for the right and left forelimbs. These served as simple kinematic indices of extension and range of motion (higher scores = better; see Supplementary Figure S1).
- Owner palatability: Assessed an owner-reported ordinal measure of supplement acceptability (lower scores = better).

Primary paired analyses compared V3 vs. V1 for each endpoint using standardized case-report forms completed immediately after each exam or ride. When feasible, the same assessor evaluated the same horse at both visits. V2 values, when present, were summarized descriptively and were not included in hypothesis testing. Additionally, a subset of two horses underwent paired radiography at V1 and V3 (see Section 2.5).

### 2.4 Statistical analysis

#### 2.4.1 General approach and software

This study was an exploratory pilot (*n* = 10 horses) designed to evaluate feasibility and tolerability and to estimate effect sizes and variability for a future randomized controlled trial. Analyses relied on paired complete cases (horses with both V1 and V3 measures) without imputation. All procedures were performed in Python (ver. 3.11) using the SciPy ecosystem, with a fixed random seed (20250810) for reproducibility: pandas (ver. 2.2), NumPy (ver. 1.26), SciPy (ver. 1.12), statsmodels (ver. 0.14), and matplotlib (ver. 3.8). Given the exploratory design, *p*-values are presented without multiplicity adjustment.

#### 2.4.2 Frequentist methods

Distinct testing strategies were applied based on data type:

- Ordinal endpoints (lameness, rider efficacy, palatability): Hypothesis testing used the paired Wilcoxon signed-rank test (exact, two-sided *α* = 0.05). Effect sizes were estimated using the Hodges–Lehmann (HL) median paired difference and the rank-biserial correlation (*r*) with bootstrap 95% confidence intervals (CIs).
- Continuous endpoints (joint angles): A sign-flip permutation test was applied on the mean paired difference (two-sided; ≥ 100,000 flips). A paired *t*-test was added as a sensitivity analysis only when differences satisfied normality assumptions (Shapiro–Wilk *p* > 0.10). Effect sizes were estimated using the mean paired difference and Hedges’ *g* (small sample corrected).

#### 2.4.3 Bayesian estimation

To complement frequentist testing, we performed Bayesian estimation on paired differences using non-informative priors. This approach allowed us to calculate the posterior mean difference, 95% credible intervals (CrI), and the probability of benefit (Pr). The probability of benefit was defined as the posterior probability that the true difference shifted in the therapeutic direction (e.g., *μ* > 0 for joint angle). Detailed mathematical specifications for the priors and posterior distributions are provided in the Supplementary methods.

#### 2.4.4 Sensitivity analysis

Sensitivity checks included a sign test for ordinal endpoints (to handle ties conservatively), a 20% trimmed-mean permutation for continuous endpoints, and leave-one-horse-out influence analysis.

### 2.5 Exploratory radiography

Radiography was prespecified as an exploratory imaging adjunct to assess the feasibility of a joint-space width (JSW) analysis pipeline and to provide anatomical context for distal limb pathology; it was not treated as a primary structural outcome. Paired radiographs (baseline and week 12) were processed using a predefined, quality-gated pipeline involving feature-based registration, region-of-interest (ROI) propagation, contrast normalization, and edge-derived JSW estimation (see Supplementary Figure S1). Following strict quality control based on view similarity (SSIM) and edge coverage, a subset of two paired datasets was retained for descriptive reporting. Technical details are provided in the Supplementary methods.

## 3 Results

### 3.1 Study population

Ten Warmblood horses with chronic lameness completed baseline (V1) and week-12 (V3) assessments. Unless noted, *n* = 10 paired observations were available per endpoint, and the walk–trot composite lameness had *n* = 9 pairs. As shown in Table 2, the mean age was 14.9 ± 2.18 years, and all were male. Per-endpoint paired analyses used complete cases (*n* listed in Table 2). Throughout the duration of the study, baseline limb/joint diagnoses and subsequent clinical notes by the examining veterinarian are provided in Supplementary Table S1.

**Table 2:** Result summaries of paired change (Δ = V3 − V1) with 95% CIs, permutation *p*, standardized effects, and Bayesian*.

Endpoint		*n* pairs	Estimate Δ (V3 − V1)	95% CI		Standardized effect (paired)	Permutation *p*	Bayesian mean Δ	95% CrI		Posterior Pr (Benefit)
				Low	High				Low	High	
Rider efficacy (grades)**	Walk	10	−0.50	−0.80	−0.20	−0.867	0.063	−0.50	−0.877	−0.123	0.993
	Trot	10	−0.50	−0.80	−0.20	−0.867	0.063	−0.50	−0.877	−0.123	0.993
Lameness (grades)	Rest	10	0	0	0	0	1.000	0	0	0	0.500
	Walk–Trot composite	9	−0.11	−0.33	0	−0.301	1.000	−0.11	−0.367	0.145	0.827
Joint angle (°)	Right	10	3.06	0.65	5.67	0.636	0.047	3.06	−0.086	6.206	0.972
	Left	10	−0.23	−1.95	1.37	−0.072	0.794	−0.23	−2.312	1.852	0.404
Owner palatability (points)		10	−1.50	−1.80	−1.20	−2.602	0.002	−1.50	−1.877	−1.123	1.000

* Δ = V3 − V1. Negative Δ indicates improvement for ordinal scales (rider efficacy, lameness, palatability); positive Δ indicates improvement for joint-angle outcomes. Confidence intervals are nonparametric bootstrap; permutation p from sign-flip test; standardized effect is paired (Hedges g with small-sample correction). Bayesian prior is non-informative for (μ, σ); Posterior Pr(benefit) uses the benefit direction shown.

** Rider-efficacy grades at walk and trot were identical within each horse at both V1 and V3; therefore, Δ (V3 − V1) vectors are identical and all statistics coincide across gaits.

### 3.2 Primary paired analyses (V3 vs. V1)

**Figure 1:**
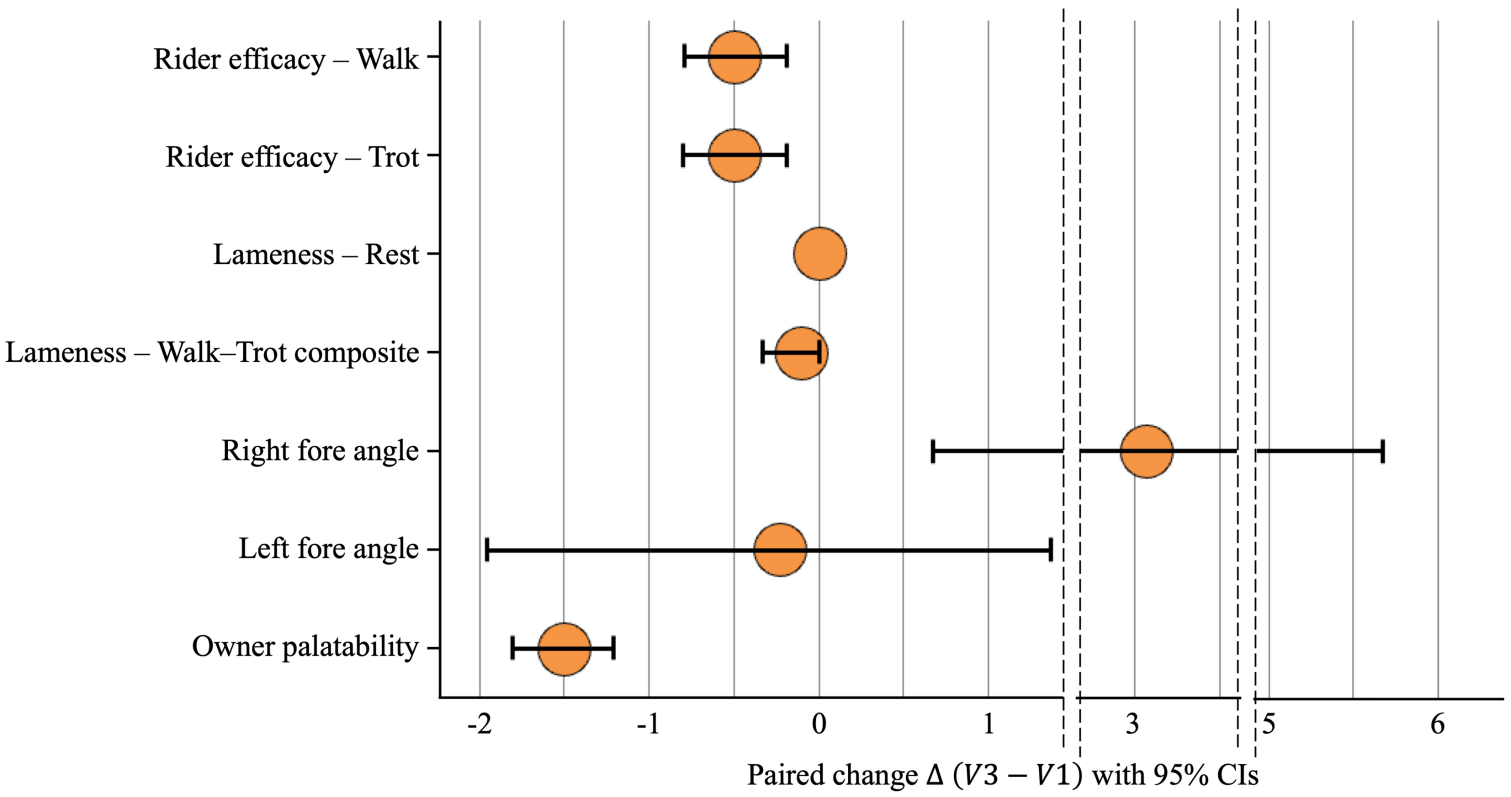
Forest plot of paired change Δ with 95% CIs across outcomes (see Table 2 for values).

**Figure 2:**
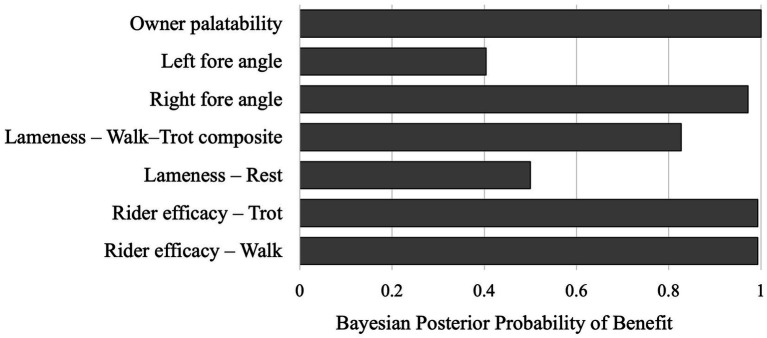
Posterior probability of benefit across outcomes (see Table 2 for values).

Paired change (Δ = V3 − V1) with 95% CIs, sign-flip permutation *p*-values, standardized paired effects, and Bayesian summaries are presented in Table 2 and visualized in Figures 1, 2.

#### 3.2.1 Rider-reported function

At walk, rider efficacy improved by Δ = −0.50 grades (95% CI − 0.80 to −0.20; standardized effect −0.867; permutation *p* = 0.063; Bayesian mean = −0.50, 95% CrI − 0.877 to −0.123; Pr(benefit) = 0.993). At trot, rider efficacy likewise improved by Δ = −0.50 (95% CI − 0.80 to −0.20; standardized effect −0.867; *p* = 0.063; Bayesian mean = −0.50, CrI − 0.877 to −0.123; Pr(benefit) = 0.993). These consistent, moderate-to-large, standardized effects indicate clinically meaningful improvements despite small-sample *p*-values hovering around the 0.05 threshold (See Table 2 and Figures 1, 2).

#### 3.2.2 Examiner-graded lameness

Examiner assessments were stratified by activity level:

- Resting lameness (*n* = 10): No change was observed across the study period (Δ = 0.00), as all scores remained stable.
- Walk–trot composite (*n* = 9): Scores trended toward improvement (Δ = −0.11, 95% CI − 0.33 to 0.00). While the frequentist analysis was statistically non-significant (*p* = 1.000), Bayesian analysis indicated a favorable probability of benefit (Mean = −0.11, Pr = 0.827), though the credible interval crossed zero (CrI − 0.367 to 0.145).

Overall, examiner-graded changes were small and imprecise, consistent with the limitations of pilot data on ordinal scales (See Table 2 and Figure 1). Narrative veterinarian findings are provided in Supplementary Table S1 to contextualize these quantitative scores.

#### 3.2.3 Joint-angle kinematics

Kinematic analysis was stratified by limb to isolate biomechanical responses because joint-angle findings differed by limb.

- Right forelimb: The joint angle increased by +3.06° (95% CI + 0.65 to +5.67, standardized effect +0.636, *p* = 0.047), consistent with a moderate improvement in range of motion. Bayesian analysis reinforced this finding (Mean = +3.06°, CrI − 0.086 to +6.206) with a high probability of benefit (Pr = 0.972).
- Left forelimb: No clear change was observed (Δ = −0.23°, 95% CI − 1.95 to +1.37, standardized effect −0.072, *p* = 0.794). Bayesian estimates remained equivocal (Mean = −0.23°, CrI − 2.312 to +1.852, Pr = 0.404).

See Table 2 and Figures 1, 2 for complete kinematic summaries.

#### 3.2.4 Acceptability

Owner-rated palatability improved markedly (Δ = −1.50 points, 95% CI − 1.80 to −1.20, standardized effect −2.602, *p* = 0.002). Bayesian analysis confirmed a definitive improvement (Mean = −1.50, CrI − 1.877 to −1.123, Pr(benefit) = 1.000). These results indicate excellent acceptability and feasibility of daily NEM dosing for 12 weeks under field conditions (See Table 2 and Figures 1, 2).

### 3.3 Exploratory radiography

Prespecified quality gates were applied to paired radiographs to ensure comparable positioning and view quality (see Supplementary methods). Only two horse-limb pairs (H02 left fore; H03 right fore) were processed and retained for descriptive JSW analysis. In both cases, apparent JSW within the predefined region of interest was wider at week 12 (V3) than at baseline (V1), ranging from approximately 4 mm to 9 mm. However, one pair showed higher view similarity and more complete edge coverage than the other. Full quality metrics and numeric JSW values are summarized in Supplementary Table S1, with representative images in Supplementary Figures S1, S2.

### 3.4 Interpretation

To clarify the hierarchy of evidence observed in this cohort, the study results were categorized by signal strength:

- Strong signals: The most consistent benefits were observed for rider-perceived under-saddle function (at both walk and trot) and owner acceptability. Bayesian posterior probabilities (Table 2; Figure 2) strongly reinforce the signal for these rider-reported outcomes.
- Moderate signal: Objective kinematic analysis showed moderate improvement in right-fore joint angle, which was also supported by the Bayesian analysis as a likely positive effect.
- Weak or equivocal signals: Examiner-graded lameness changes were small and imprecise, a result expected given the sample size (*n* = 10) and the limitations of ordinal scales. Similarly, evidence for changes in the left-fore angle and resting lameness remained equivocal.

## 4 Discussion

### 4.1 Principal findings

In this 12-week, single-arm pilot of Warmblood horses with chronic lameness, rider-reported under-saddle function improved by approximately one-half grade at both walk and trot (Δ = V3 − V1 ≈ −0.50 for each gait; lower is better), accompanied by a moderate increase in right-fore joint angle (Δ ≈ +3.1°, higher is better) and a large improvement in owner-reported palatability (Δ ≈ −1.5 points). Examiner-graded lameness at rest and a walk–trot composite showed small, imprecise changes. Bayesian posterior probabilities of benefit were high for rider-perceived function (≈0.99) and right-fore kinematics (≈0.97), equivocal for left-fore angle (≈0.40) and rest lameness (0.50). Together, these patterns indicate good tolerability and feasibility, with the strongest signal in rider-perceived under-saddle function and supportive improvement in a simple kinematic measure.

### 4.2 Interpretation and clinical relevance

Standard AAEP-aligned lameness grades provide a necessary framework for communication, but they are ordinal, observer-dependent, and often insensitive to modest functional change, particularly in small samples with mild pathology (5, 6). Previous studies have shown only partial agreement between visual grading and quantitative gait asymmetry, emphasizing the prevalence of discordance between these methods. Consequently, reliance on visual grading alone may miss subtle improvements (6, 20, 21). These data support our strategy of triangulating rider-reported function with objective measures to capture day-to-day function and range of motion that may not be captured by small shifts in examiner-assigned AAEP grades (5, 7).

Within that framework, the approximately half-grade improvement in rider-reported function at both walk and trot gaits, observed by the same riders under field conditions, represents a change that is likely to be meaningful for training decisions and perceived comfort, even if frequentist *p*-values are near the 0.05 thresholds in a cohort of 10 horses. Furthermore, the ~3° increase in right-fore joint angle indicates an objective improvement in range of motion (reduced stiffness) on the most responsive limb. This is consistent with the mechanistic concept that early benefits of joint-directed interventions may manifest primarily as functional or symptomatic improvements before substantial structural differences are detectable radiographically over short intervals (5–7).

Finally, clinical relevance relies on long-term adherence. Palatability improved markedly in this study, a critical finding given that supplement acceptability is the primary predictor of owner compliance in longer programs. High acceptability also minimizes the confounding risk that poor tolerability or refusal of the supplement masked a potential therapeutic signal.

### 4.3 Relation to prior work on nutraceuticals and NEM

NEM provides joint-relevant macromolecules (collagen, glycosaminoglycans, hyaluronic acid) and exhibits anti-inflammatory and chondroprotective effects on OA-related pathways implicated in osteoarthritis pain and stiffness (8–12, 14). Randomized and controlled human studies have reported clinically relevant reductions in pain and stiffness within weeks of NEM supplementation, and veterinary studies in dogs and experimental models have shown improvements in mobility/quality-of-life and chondroprotection with eggshell-membrane–containing formulations, suggesting functional joint benefits across species (8, 10–18, 22). Equine-specific data remain limited, but prior works in various species utilizing NEM support translational plausibility (18). Our observation of rider-perceived function with a modest, supportive kinematic signal is consistent with these prior works and with the broader nutraceutical literature in veterinary orthopedics, where earlier changes are also observed in comfort and function than in structural imaging endpoints (8, 10, 16).

### 4.4 Radiography as exploratory context

Short-interval structural change on plain radiographs is challenging to establish reliably in equine distal joints because apparent joint-space width (JSW) is highly sensitive to beam angle, limb stance, and rotation (7, 23). Even with careful positioning and quality control, modest differences in projection can alter the apparent distance between opposing subchondral bone margins and thereby mimic or obscure true change. For this reason, radiography was prespecified as an exploratory adjunct in this pilot rather than a primary structural endpoint.

In practice, usable paired JSW measurements were obtained for only two horses, reflecting the stringent requirements for comparable positioning and view quality over time. Both pairs showed small apparent widenings of JSW, accompanied by changes in regional radiographic density, but the direction and magnitude of these differences were within a range that can plausibly be explained by variation in limb stance and projection at the time of imaging. Given the very small number of analyzable pairs and the strong dependence of JSW on imaging geometry, these radiographic findings cannot be interpreted as evidence of structural modification and must be regarded as hypothesis-generating only.

The absence of a decisive radiographic signal over 12 weeks does not contradict the rider-reported functional improvements observed in this study. Rather, it underscores that plain-film JSW is a technically fragile surrogate for structural change over short intervals in field conditions and that functional outcomes are likely to be more sensitive to early symptomatic effects. We therefore present the radiographic analyses primarily to illustrate a feasible imaging and analysis pipeline for future, larger controlled trials, where standardized positioning, longer follow-up, and greater sample size will be required to evaluate potential structural effects more robustly (Supplementary Table S2; Supplementary Figure S2).

### 4.5 Strengths and limitations

Strengths of this study include a prospective paired design, prespecified endpoints spanning examiner, rider, kinematic, and acceptability domains. Additionally, small-sample–appropriate statistics (exact/permutation tests, bootstrap CIs, standardized paired effects, and Bayesian estimation) also strengthen this study. However, several limitations should be acknowledged. The cohort was small (*n* = 10) and consisted only of male horses, and the uncontrolled design limits causal inference. Rider and owner assessments were unblinded, introducing potential expectation and reporting bias; we attempted to mitigate this by using the same rider per horse, focusing on within-horse changes, and triangulating rider reports with examiner grading and kinematic measures, but residual bias cannot be excluded. In addition, multiplicity was not adjusted in this exploratory setting, so emphasis is placed on effect sizes and intervals rather than *p*-values. Finally, the lack of inertial sensor-based gait asymmetry and standardized radiographic positioning constrains objective triangulation with examiner grades.

### 4.6 Implications and future directions

These pilot data support the feasibility and acceptability of 12-week NEM dosing in lame Warmblood horses and indicate a promising functional signal that merits a confirmatory trial. A parallel-group, randomized, blinded, placebo-controlled study would best address expectation effects and regression to the mean. We recommend prespecifying a single primary endpoint among rider-reported under-saddle function (walk or trot) or an objective kinematic metric (e.g., inertial sensor asymmetry or validated joint-angle measure), with the other domains as secondary endpoints to limit multiplicity.

Taken together, the results suggest a promising functional signal that warrants a randomized, blinded, placebo-controlled trial in horses with naturally occurring OA. Elements to prioritize include a single primary endpoint to limit multiplicity (either rider-reported under-saddle function at a prespecified gait or a validated objective kinematic measure) and stratification by baseline severity and affected limb. Additionally, it also includes centralized rater training and consistent surfaces, standardized farriery/exercise, and instrumented gait (e.g., inertial sensors) to complement visual grading. These recommendations are consistent with contemporary OA management frameworks that emphasize multimodal assessment and functional endpoints in veterinary practice (5, 7, 24). Incorporating inertial sensor gait analysis and standardized radiography (including positioning rigs and calibration) would strengthen mechanistic interpretation. The effect sizes and variances estimated here can inform sample-size planning, given the observed ~0.5-grade paired change and variability, a substantially larger cohort will be needed to reliably detect group differences with adequate power.

## 5 Conclusion

In this 12-week, prospective before–after pilot of Warmblood horses with chronic lameness, daily oral NEM (12 mg/kg) was feasible and well tolerated, with coherent functional benefits. Rider-reported under-saddle function improved by half a grade at walk and trot, right-fore joint angle increased, and owner palatability improved markedly. Examiner-graded lameness showed small, imprecise group-level changes, which were expected with an ordinal scale and a small sample size. Bayesian posteriors reinforced the functional pattern (high probability of benefit for rider scores and right-fore kinematics).

These data do not establish causality, but they support proceeding to a randomized, blinded, placebo-controlled trial. A confirmatory study should prespecify one primary endpoint (rider function at a single gait or an objective locomotor metric), use stratification by baseline severity/affected limb, standardize surfaces and rater training, log adherence, and add instrumented gait analysis. Radiography should follow standardized positioning and serve as a longer-term structural outcome. The effect sizes observed in this pilot provide practical inputs for sample-size planning.

## Data Availability

The raw data supporting the conclusions of this article will be made available by the authors, without undue reservation.

## References

[1] Baccarin RYA Seidel SRT Michelacci YM Tokawa PKA Oliveira TM. Osteoarthritis: a common disease that should be avoided in the athletic horse's life. Anim Front. (2022) 12:25–36. doi: 10.1093/af/vfac026, 35711506 PMC9197312

[2] Mayet A Zablotski Y Roth SP Brehm W Troillet A. Systematic review and Meta-analysis of positive long-term effects after intra-articular Administration of Orthobiologic Therapeutics in horses with naturally occurring osteoarthritis. Front Vet Sci. (2023) 10:1125695. doi: 10.3389/fvets.2023.1125695, 36908512 PMC9997849

[3] Estrada McDermott J Pezzanite L Goodrich L Santangelo K Chow L Dow S . Role of innate immunity in initiation and progression of osteoarthritis, with emphasis on horses. Animals. (2021) 11:113247. doi: 10.3390/ani11113247, 34827979 PMC8614551

[4] AAEP. (2025) Client education presentation: Lameness exams. Available online at: https://aaep.org/resource/client-education-presentation-lameness-exams/. [Accessed May 1, 2025]

[5] Davidson EJ. Lameness evaluation of the athletic horse. Vet Clin North Am Equine Pract. (2018) 34:181–91. doi: 10.1016/j.cveq.2018.04.013, 30007446

[6] Hardeman AM Egenvall A Serra Braganca FM Swagemakers JH Koene MHW Roepstorff L . Visual lameness assessment in comparison to quantitative gait analysis data in horses. Equine Vet J. (2022) 54:1076–85. doi: 10.1111/evj.13545, 34913524 PMC9786350

[7] Trencart P Alexander K De Lasalle J Laverty S. Radiographic evaluation of the width of the femorotibial joint space in horses. Am J Vet Res. (2016) 77:127–36. doi: 10.2460/ajvr.77.2.127, 27027705

[8] Ruff KJ DeVore DP Leu MD Robinson MA. Eggshell membrane: a possible new natural therapeutic for joint and connective tissue disorders. Results from two open-label human clinical studies. Clin Interv Aging. (2009) 4:235–40. doi: 10.2147/cia.s5797, 19554094 PMC2697588

[9] Jia H Hanate M Aw W Itoh H Saito K Kobayashi S . Eggshell membrane powder ameliorates intestinal inflammation by facilitating the restitution of epithelial injury and alleviating microbial dysbiosis. Sci Rep. (2017) 7:43993. doi: 10.1038/srep43993, 28272447 PMC5341015

[10] Hewlings S Kalman D Schneider LV. A randomized, double-blind, placebo-controlled, prospective clinical trial evaluating water-soluble chicken eggshell membrane for improvement in joint health in adults with knee osteoarthritis. J Med Food. (2019) 22:875–84. doi: 10.1089/jmf.2019.0068, 31381494 PMC6748399

[11] Kim JW Lee DH Lee KW Na IS Lee NY Kim JK . Profiling bioactive components of natural eggshell membrane (Nem) for cartilage protection and its protective effect on oxidative stress in human chondrocytes. Int J Mol Sci. (2024) 25:11304. doi: 10.3390/ijms252011304, 39457086 PMC11508478

[12] Kiers JL Bult JHF. Mildly processed natural eggshell membrane alleviates joint pain associated with osteoarthritis of the knee: a randomized double-blind placebo-controlled study. J Med Food. (2021) 24:292–8. doi: 10.1089/jmf.2020.0034, 32633648 PMC7989856

[13] Ruff KJ Winkler A Jackson RW DeVore DP Ritz BW. Eggshell membrane in the treatment of pain and stiffness from osteoarthritis of the knee: a randomized, multicenter, double-blind, placebo-controlled clinical study. Clin Rheumatol. (2009) 28:907–14. doi: 10.1007/s10067-009-1173-4, 19340512 PMC2711914

[14] Kim J-I Choi J-H Seo M-S Kim J-K Chun Y-S Kwon Y-S . Natural eggshell membrane attenuates chondrocyte inflammation and surgically induced osteoarthritis in rats. Appl Sci. (2024) 14:5176. doi: 10.3390/app14125176

[15] Ragetly GR Martins A Ober CA Boiocchi S Nicolas CS. Efficacy of a joint supplement containing eggshell membrane among other ingredients to improve the mobility of dogs with osteoarthritis: a Multicenter double-blind randomized placebo-controlled study. Front Vet Sci. (2025) 12:1561793. doi: 10.3389/fvets.2025.1561793, 40530040 PMC12171436

[16] Ruff KJ Kopp KJ Von Behrens P Lux M Mahn M Back M. Effectiveness of Nem((R)) brand eggshell membrane in the treatment of suboptimal joint function in dogs: a multicenter, randomized, double-blind, placebo-controlled study. Vet Med. (2016) 7:113–21. doi: 10.2147/VMRR.S101842, 30050844 PMC6044796

[17] Ruff KJ Morrison D Duncan SA Back M Aydogan C Theodosakis J. Beneficial effects of natural eggshell membrane versus placebo in exercise-induced joint pain, stiffness, and cartilage turnover in healthy, postmenopausal women. Clin Interv Aging. (2018) 13:285–95. doi: 10.2147/CIA.S153782, 29497287 PMC5822842

[18] Wedekind KJ Coverdale JA Hampton TR Atwell CA Sorbet RH Lunnemann J . Efficacy of an equine joint supplement, and the synergistic effect of its active ingredients (chelated trace minerals and natural eggshell membrane), as demonstrated in equine, swine, and an osteoarthritis rat model. Open Access Animal Physiol. (2015) 7:7–13. doi: 10.2147/OAAP.S75022

[19] Adenot CC Abdelhakim HE. Palatability assessment of oral dosage forms for companion animals: a systematic review. J Drug Deliv Sci Technol. (2022) 77:103841. doi: 10.1016/j.jddst.2022.103841

[20] Keegan KG Kramer J Yonezawa Y Maki H Pai PF Dent EV . Assessment of repeatability of a wireless, inertial sensor-based lameness evaluation system for horses. Am J Vet Res. (2011) 72:1156–63. doi: 10.2460/ajvr.72.9.1156, 21879972

[21] Keegan KG Wilson DA Kramer J Reed SK Yonezawa Y Maki H . Comparison of a body-mounted inertial sensor system-based method with subjective evaluation for detection of lameness in horses. Am J Vet Res. (2013) 74:17–24. doi: 10.2460/ajvr.74.1.17, 23270341

[22] Muller C Enomoto M Buono A Steiner JM Lascelles BDX. Placebo-controlled pilot study of the effects of an eggshell membrane-based supplement on mobility and serum biomarkers in dogs with osteoarthritis. Vet J. (2019) 253:105379. doi: 10.1016/j.tvjl.2019.105379, 31685140

[23] Contino EK Barrett MF Werpy NM. Effect of limb positioning on the radiographic appearance of the distal and proximal interphalangeal joint spaces of the forelimbs of horses during evaluation of Dorsopalmar radiographs. J Am Vet Med Assoc. (2014) 244:1186–90. doi: 10.2460/javma.244.10.1186, 24786167

[24] Mosley C Edwards T Romano L Truchetti G Dunbar L Schiller T . Proposed Canadian consensus guidelines on osteoarthritis treatment based on OA-coast stages 1-4. Front Vet Sci. (2022) 9:830098. doi: 10.3389/fvets.2022.830098, 35558892 PMC9088681

