# Supplementary material for: Natural eggshell membrane supplementation for chronic lameness in warmblood horses: a 12-week prospective before–after study
Source: Front Vet Sci. 2026 Feb 26;13:1711135. doi: 10.3389/fvets.2026.1711135 (PMC12980879; doi:10.3389/fvets.2026.1711135)
Supplement: Supplementary file 1 [file Data_Sheet_1.pdf]

Supplementary Material

1 Supplementary Methods

Statistical analysis: Bayesian framework for paired differences

To estimate the probability of therapeutic effects, we employed a Bayesian approach using non-informative priors. This method provides a probabilistic interpretation of the parameter estimates (mean difference) without relying on large-sample asymptotic assumptions.

For each endpoint, let $\Delta = x_{V3} - x_{V1}$ represent the vector of paired differences for the n complete cases. We assumed the differences follow a normal distribution:

$$\Delta \sim N(\mu, \sigma^2)$$

We utilized the standard Jeffreys non-informative prior for the unknown mean (μ) and variance (σ^2):

$$p(\mu, \sigma^2) \propto \frac{1}{\sigma^2}$$

Under this prior, the marginal posterior distribution for the mean difference μ follows a Student's *t*-distribution:

$$\mu | \Delta \sim t_{n-1}\left(\bar{\Delta}, \frac{s}{\sqrt{n}}\right)$$

Where:

- n is the number of pairs,
- $\bar{\Delta}$ is the sample mean of the differences,
- s is the sample standard deviation of the differences.

The probability of benefit reported is the integral of the posterior density over the region of therapeutic interest.

- For "higher-is-better" outcomes (e.g., Joint Angle, Palatability):

$$\Pr(benefit) = \int_0^{\infty} p(\mu|\Delta) d\mu$$

- For "lower-is-better" outcomes (e.g., Lameness Scores):

$$\Pr(benefit) = \int_{-\infty}^{0} p(\mu|\Delta)d\mu$$

This metric represents the probability that the true population mean difference is greater than zero (or less than zero) given the observed data.

Fetlock joint range-of-motion (ROM) measurement

In this study, range of motion (ROM) was consistently evaluated at the fetlock (metacarpophalangeal) joint using a standardized measurement site. This choice was based on anatomical and clinical grounds: all enrolled horses had pain lesions localized to the distal forelimb. As summarized in Supplementary Table S1, veterinary findings included suspected coffin joint inflammation, navicular apparatus changes, pastern joint lesions, and fetlock-associated pain. These distal pathologies were expected to exert the greatest biomechanical impact on the fetlock during locomotion.

As shown in Supplementary Figure S1, ROM assessment was performed by flexing the forelimb and measuring the maximal flexion angle around the fetlock joint with a digital goniometer. The anatomical landmarks and flexion axis visible in the photograph correspond to the metacarpophalangeal (fetlock) joint. Supplementary Figure S2 shows radiographs centered on the coffin bone, navicular bone, and pastern region, emphasizing that lesions in this cohort were concentrated along the coffin–navicular–pastern axis. This lesion distribution directly alters the flexion–extension pattern at the fetlock and provides a mechanistic basis for using fetlock ROM as a sensitive functional indicator in chronic distal limb pain.

ROM evaluation was performed on horses standing square on a flat surface. A trained examiner gently lifted the forelimb and induced passive flexion centered on the fetlock. The digital goniometer was aligned anatomically along the third metacarpal bone (MC3) proximally and the proximal phalanx distally, with the axis of rotation consistently at the fetlock joint. Flexion was advanced to a firm but comfortable end-feel without eliciting obvious pain. Measurements were repeated twice per limb and averaged for analysis.

ROM was assessed by a single experienced examiner who closely monitored for pain responses and stopped at a "firm but non-painful end-feel"; forced maximal flexion was not applied. In all horses, discomfort was minimal to absent, and pain-avoidance behaviors were not observed. This standardized end-feel approach was considered clinically appropriate and ethically justified for functional evaluation of distal forelimb lesions (coffin, pastern, navicular) and was performed under the oversight of an independent veterinarian and the owner.

Fetlock was chosen rather than carpus because all lesions in the enrolled horses were localized to the distal forelimb (coffin joint, pastern joint, navicular apparatus) rather than the carpal joint. Chronic distal limb osteoarthritis/navicular pain is most sensitively reflected as changes in fetlock ROM, particularly reduced flexion and end-range stiffness, and our assessments were focused on this distal-limb axis (Supplementary Figures S1–S2). Therefore, fetlock ROM was judged to be the most clinically and biomechanically appropriate joint for this study's objectives.

Force plates and inertial-sensor systems are valuable objective tools but are mainly used in laboratory or referral settings to evaluate whole-limb symmetry or ground-reaction forces. In contrast, the lesions observed in this study were localized to the distal forelimb, where mechanical changes such as reduced fetlock flexion and end-range stiffness are most sensitive to pain. For that reason, fetlock ROM was used as a pragmatic, lesion-targeted functional index suitable for field-based, longer-term follow-up, rather than as a replacement for laboratory-based force-plate analysis.

Exploratory radiography

A subset of two horses underwent paired radiography at V1 and V3 for exploratory structural assessment. Radiographs were rigidly registered to compare like-for-like anatomy, a joint region of interest (ROI) was propagated from baseline to follow-up, and apparent joint space width (JSW) was estimated by edge detection. All processing was performed in Python (ver. 3.11) using OpenCV (ver. 4.9), scikit-image (ver. 0.22), and NumPy (ver. 1.26). Images were converted to 8-bit grayscale, resampled to a common field (longest side 2048 px when larger), and intensity-normalized with CLAHE (clipLimit 2.0, tileGridSize 8×8). Each V3 image was registered to its V1 counterpart using ORB keypoints with Hamming matching and RANSAC inlier selection, followed by a similarity transform (rotation, uniform scale, translation); phase-correlation provided a translation fallback when features were insufficient. Registration quality was summarized by the structural similarity index (SSIM) over the full image and within the joint ROI.

An analyst defined a rectangular ROI on V1 to enclose the articular compartment of interest; this ROI was propagated to V3 via the registration transform and eroded by 5–10 px to avoid collimation borders and labels. When a radiopaque calibration object or DICOM pixel spacing was available, measurements were reported in millimeters; otherwise, values were reported in pixels and explicitly labeled as apparent JSW. Within each ROI, JSW was estimated as the distance between opposing subchondral bone edges along lines approximately perpendicular to the joint line after Gaussian denoising (5×5 kernel, $\sigma = 1.0$), gradient and edge extraction (Sobel magnitude and Canny with thresholds initialized by the median absolute deviation and refined to retain strong, coherent edges), and joint-line orientation estimation by principal-component analysis of high-gradient pixels with rotation to make the joint line approximately horizontal. Superior and inferior subchondral boundaries were traced as the strongest contiguous edge ridges within bands 15–25 px above and below the joint line, followed by morphological closing (3×3) and spur removal. For each image column, the vertical distance between boundaries yielded a per-column JSW profile; the median across valid columns served as the primary JSW summary, and coverage was defined as the percentage of ROI columns with valid paired edges.

To reduce projection and stance bias, pairs were included in ΔJSW summaries only when SSIM within the ROI ≥ 0.75 and coverage ≥ 80%. Views that failed quality criteria were illustrated with overlays and excluded from ΔJSW summaries. For views meeting quality criteria, we reported JSW_V1, JSW_V3, and ΔJSW = JSW_V3 − JSW_V1 (widening > 0; narrowing < 0), with SSIM and coverage as accompanying quality metrics. Inference remained descriptive but was complemented by small-sample procedures: bootstrap 95% CIs for ΔJSW (2000 resamples; seed 20250810), a paired sign-flip permutation test on mean ΔJSW (two-sided; 100,000 flips; seed 20250810), and a Bayesian posterior for mean ΔJSW under a Jeffreys prior for (μ, σ), summarized by the posterior mean, 95% CrI, and Pr($\mu > 0$). Imaging analyses were performed blinded to clinical outcomes. Given the projection dependence of plain-film JSW over short intervals, these findings are labeled non-confirmatory and are presented

only as Supplementary with a result summary (Supplementary Table S2) and sample X-ray images (Supplementary Figure S2).

2 Supplementary Figures and Tables

Table S1. Veterinarian clinical findings at baseline (V1), week 4 (V2; descriptive only), and week 12 (V3) for each horse.

Horse ID	Baseline, Lameness Features	Week 4, Lameness Features	Week 12, Lameness Features
H01	No specific findings	Lameness in both straight and circular trotting	No specific findings
H02	Lameness in the right forelimb	Lameness: Left forelimb coffin joint inflammation	No specific findings
	Lameness: Left forelimb coffin joint inflammation		
H03	Lameness in the left forelimb	Lameness: Right forelimb coffin joint and navicular syndrome	Right forelimb lameness and coffin joint are better
	Lameness: Right forelimb coffin joint and navicular syndrome	Lameness: Right forelimb coffin joint and navicular syndrome	No specific findings
H04	Lameness: Left forelimb ring bone	Lameness: Left forelimb ring bone	Left forelimb lameness is also better than before when viewed from a distance, but the condition is similar to before
H05	Lameness: Right forelimb medial side-bone	Lameness: Right forelimb medial side-bone	No specific findings
H06	Lameness: Right hindlimb deep digital flexor tendon strain	Lameness: Right hindlimb deep digital flexor tendon strain	No specific findings
H07	Lameness: Left forelimb fetlock joint	Lameness: Left forelimb fetlock joint	No specific findings
H08	Lameness: Right hindlimb hock joint	Lameness: Right hindlimb hock joint	No specific findings
H09	Lameness: Right forelimb navicular syndrome	Lameness: Right forelimb navicular syndrome	No specific findings
H10	Lameness: Left hindlimb medial side-bone	Lameness: Left hindlimb medial side-bone	No specific findings

* Notes: V2 assessments were collected for descriptive context and were not included in hypothesis testing. ‘No specific findings’ indicates no new abnormalities relative to prior exam.

Table S2. Analytical summary of X-ray image data.

Horse ID	side	Inlier matches	Coverage		JSW median (px)		JSW Delta (px)	Density Mean		Density Delta Mean	P90		SSIM	MSE	px per mm	JSW (mm)		
			V1	V3	V1	V3		V1	V3		V1	V3				V1	V3	Delta
H02	L	14	0.977	0.981	248	268	20	0.5292	0.5107	-0.0185	0.6588	0.6667	0.463	0.018	4.58	54.15	58.52	4.37 (+8.1%)
H03	R	8	0.953	0.415	230	269.5	39.5	0.4007	0.1579	-0.2429	0.6941	0.5608	0.113	0.105	4.44	51.8	60.7	8.9 (+17.2%)

* Notes: Values reflect apparent joint space width (JSW) derived from region of interest (ROI) edge distances and calibration (px/mm) and are projection-dependent; they do not represent anatomical joint cartilage thickness.

2.1 Supplementary Figures

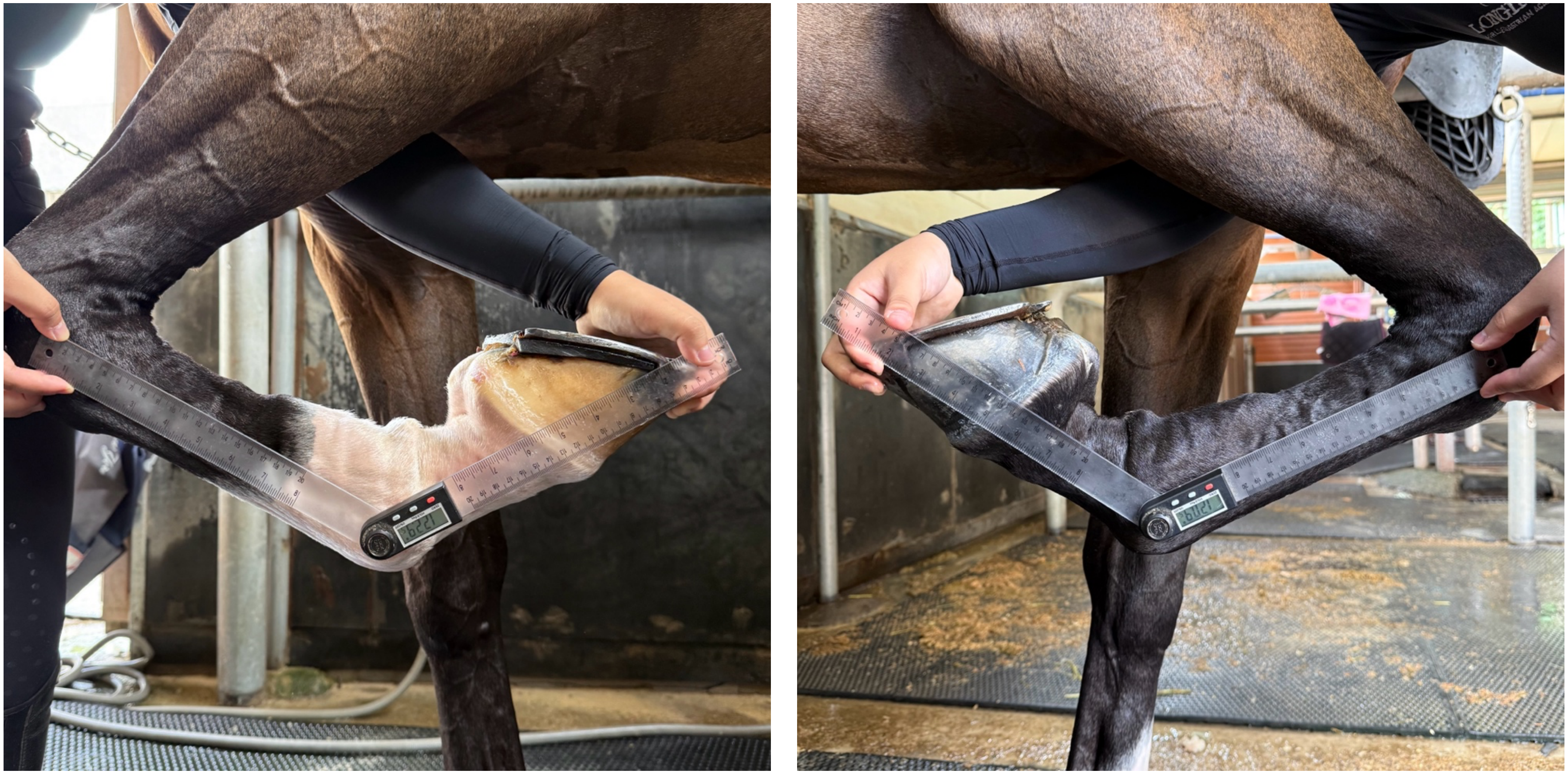

Supplementary Figure S1. Measures of joint angle. A goniometer was used to assess maximum flexion or the range of motion (ROM).

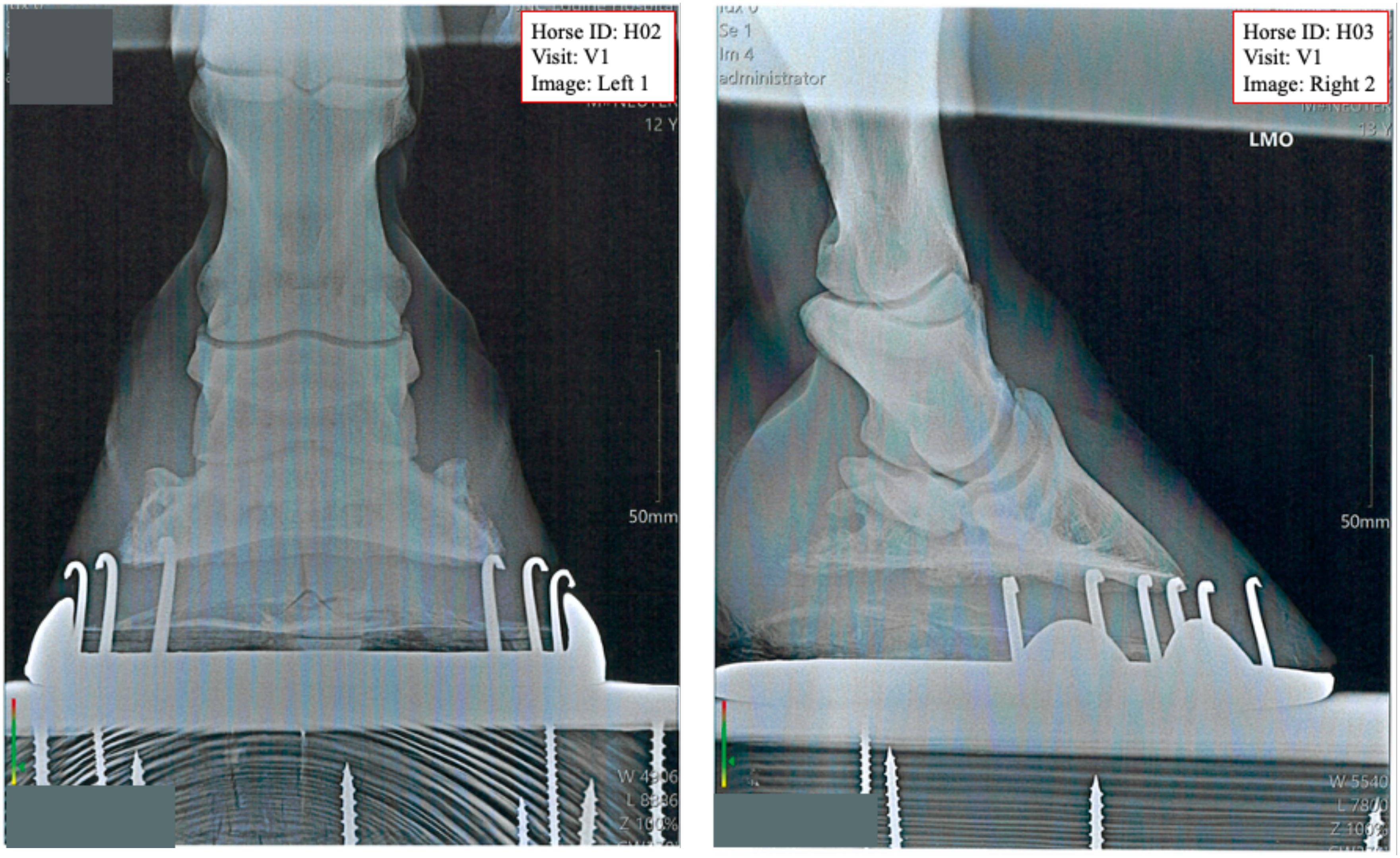

Supplementary Figure S2. X-ray sample images.
